# (*Z*)-*N*-Methyl-2-(5-methyl-2-oxoindolin-3-yl­idene)hydrazinecarbothio­amide

**DOI:** 10.1107/S1600536812005417

**Published:** 2012-03-03

**Authors:** Amna Qasem Ali, Naser Eltaher Eltayeb, Siang Guan Teoh, Abdussalam Salhin, Hoong-Kun Fun

**Affiliations:** a School of Chemical Sciences, Universiti Sains Malaysia, Minden, Penang, Malaysia; b Faculty of Science, Sabha University, Libya; c Department of Chemistry, International University of Africa, Khartoum, Sudan; d X-ray Crystallography Unit, School of Physics, Universiti Sains Malaysia, 11800 USM, Penang, Malaysia

## Abstract

In the title compound, C_11_H_12_N_4_OS, an intra­molecular N—H⋯O hydrogen bond generates an *S*(6) ring motif. In the crystal, the mol­ecules form a helical chain along the *a* axis through an N—H⋯O hydrogen bond. These chains are extended by an N—H⋯S hydrogen bond and a C—H⋯π inter­action into a three-dimensional network.

## Related literature

For related structures, see: Ali *et al.* (2012 ▶); Qasem Ali *et al.* (2012 ▶, 2011*a* ▶,*b* ▶). For various biological activities of Schiff bases, see: Bhandari *et al.* (2008 ▶); Bhardwaj *et al.* (2010 ▶); Pandeya *et al.* (1999 ▶); Sridhar *et al.* (2002 ▶); Suryavanshi & Pai (2006 ▶). For cytotoxic and anti­cancer activities of isatin and its derivatives, see: Vine *et al.* (2009 ▶). For graph-set analysis, see: Bernstein *et al.* (1995 ▶).
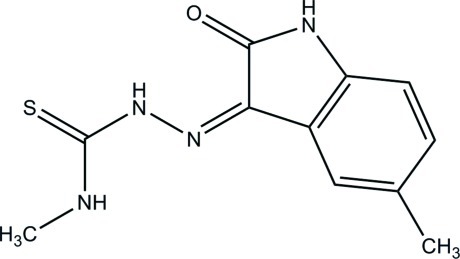


## Experimental

### 

#### Crystal data

C_11_H_12_N_4_OS
*M* *_r_* = 248.31
Orthorhombic, 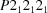

*a* = 6.2826 (2) Å
*b* = 10.0341 (3) Å
*c* = 19.1315 (5) Å
*V* = 1206.05 (6) Å^3^
*Z* = 4
Mo *K*α radiation
μ = 0.26 mm^−1^
*T* = 100 K
0.51 × 0.18 × 0.13 mm

#### Data collection

Bruker APEXII CCD diffractometer
Absorption correction: multi-scan (*SADABS*; Bruker, 2005 ▶) *T*_min_ = 0.879, *T*_max_ = 0.967
13743 measured reflections
3780 independent reflections
3463 reflections with *I* > 2σ(*I*)
*R*_int_ = 0.050

#### Refinement

*R*[*F* ^2^ > 2σ(*F* ^2^)] = 0.037
*wR*(*F* ^2^) = 0.097
*S* = 1.07
3780 reflections
168 parameters
H atoms treated by a mixture of independent and constrained refinement
Δρ_max_ = 0.32 e Å^−3^
Δρ_min_ = −0.24 e Å^−3^
Absolute structure: Flack (1983 ▶), with 1584 Friedel pairs
Flack parameter: −0.08 (7)

### 

Data collection: *APEX2* (Bruker, 2005 ▶); cell refinement: *SAINT* (Bruker, 2005 ▶); data reduction: *SAINT*; program(s) used to solve structure: *SHELXS97* (Sheldrick, 2008 ▶); program(s) used to refine structure: *SHELXL97* (Sheldrick, 2008 ▶); molecular graphics: *SHELXTL* (Sheldrick, 2008 ▶); software used to prepare material for publication: *SHELXTL* and *PLATON* (Spek, 2009 ▶).

## Supplementary Material

Crystal structure: contains datablock(s) I, global. DOI: 10.1107/S1600536812005417/is5066sup1.cif

Structure factors: contains datablock(s) I. DOI: 10.1107/S1600536812005417/is5066Isup2.hkl

Supplementary material file. DOI: 10.1107/S1600536812005417/is5066Isup3.cml

Additional supplementary materials:  crystallographic information; 3D view; checkCIF report

## Figures and Tables

**Table 1:** Hydrogen-bond geometry (Å, °) *Cg*2 is the centroid of the C1–C6 ring.

*D*—H⋯*A*	*D*—H	H⋯*A*	*D*⋯*A*	*D*—H⋯*A*
N1—H1N1⋯O1^i^	0.81 (2)	2.03 (2)	2.8319 (17)	171 (2)
N3—H1N3⋯O1	0.84 (2)	2.079 (19)	2.7525 (17)	136.9 (17)
N4—H1N4⋯S1^ii^	0.80 (2)	2.85 (2)	3.5538 (13)	148.5 (19)
C3—H3*A*⋯*Cg*2^iii^	0.95	2.62	3.4165 (16)	142

Symmetry codes: (i) 
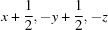
; (ii) 
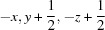
; (iii) 
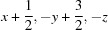
.

**Fig. 1.**
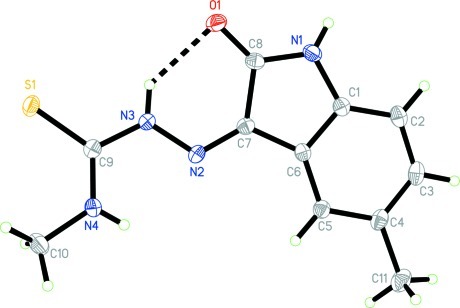
The molecular structure of the title compound, with 50% probability displacement ellipsoids and the atom-numbering scheme.

**Fig. 2.**
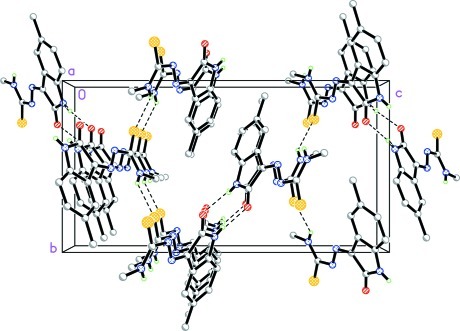
The crystal packing of the title compound viewed down the a axis. Hydrogen bonds are shown as dashed lines.

## supplementary crystallographic information

### Comment

Isatin (2,3-dioxindole) is an endogenous compound identified in humans, and its effect has been studied in a variety of systems. Biological properties of isatin and its derivatives include a range of actions in the brain, offer protection against bacterial (Suryavanshi & Pai, 2006) and fungal infections and possess anticonvulsant, anti-HIV (Pandeya *et al.*, 1999), anti-depressant and anti-inflammatory activities (Bhandari *et al.*, 2008). Recently, we reported the crystal structure of (*Z*)-*N*-methyl-2-(5-nitro-2-oxoindolin-3-ylidene) hydrazinecarbothioamide (Ali *et al.*, 2012). In the present paper we describe the single-crystal X-ray diffraction study of title compound.

In this compound (Fig. 1), the chain N2/N3/C9/S1/N4/C10 connects to the nine-membered 5-methylindolin-2-one ring system at C7. In this chain, C7/N2/N3/C9 and C10/N4/C9/S1 have torsion angles -176.69 (13) and -1.4 (2)°, respectively. The essentially planar conformation of the molecule is maintained by the cyclic intramolecular N3—H1N3···O1 hydrogen-bond (Table 1) [graph set *S*(6); Bernstein *et al.*, 1995)]. In the crystal, the molecules form a helical chain through an intermolecular N1—H1N1···O1 hydrogen bond and are extended by an N4—H1N4···S1 hydrogen bond and a weak C3—H3A···*Cg*2 interaction into a three-dimensional network (Table 1, Fig. 2). *Cg*2 is the centroid of the C1–C6 ring.

### Experimental

The Schiff base has been synthesized by refluxing the reaction mixture of hot ethanolic solution (30 ml) of 5-methyl-3-thiosemicarbazide (0.01 mol) and hot ethanolic solution (30 ml) of 5-methylisatin (0.01 mol) for 2 h. The precipitate formed during reflux was filtered, washed with cold EtOH and recrystallized from hot EtOH (yield 94%, m.p. 551.7–552.2 K). The yellow crystals were grown in acetone–DMF (3:1) by slow evaporation at room temperature.

### Refinement

N-bound H atoms were located in a difference Fourier map and were refined freely. The remaining H atoms were positioned geometrically and refined using a riding model, with C—H = 0.95 Å for aromatic ring and C—H = 0.98 Å for methyl group, and with *U*_iso_(H) = 1.2*U*_eq_(C) and 1.5*U*_eq_(C) for aromatic ring and methyl group, respectively. The highest residual electron density peak is located at 0.76 Å from C9 and the deepest hole is located at 0.16 Å from H11C.

### Crystal data

**Table:**

C_11_H_12_N_4_OS	*D*_x_ = 1.368 Mg m^−^^3^
*M**_r_* = 248.31	Melting point = 551.7–552.2 K
Orthorhombic, *P*2_1_2_1_2_1_	Mo *K*α radiation, λ = 0.71073 Å
Hall symbol: P 2ac 2ab	Cell parameters from 5511 reflections
*a* = 6.2826 (2) Å	θ = 2.3–30.7°
*b* = 10.0341 (3) Å	µ = 0.26 mm^−^^1^
*c* = 19.1315 (5) Å	*T* = 100 K
*V* = 1206.05 (6) Å^3^	Block, orange
*Z* = 4	0.51 × 0.18 × 0.13 mm
*F*(000) = 520	

### Data collection

**Table:**

Bruker APEXII CCD diffractometer	3780 independent reflections
Radiation source: fine-focus sealed tube	3463 reflections with *I* > 2σ(*I*)
Graphite monochromator	*R*_int_ = 0.050
φ and ω scans	θ_max_ = 31.0°, θ_min_ = 2.1°
Absorption correction: multi-scan (*SADABS*; Bruker, 2005)	*h* = −8→9
*T*_min_ = 0.879, *T*_max_ = 0.967	*k* = −14→13
13743 measured reflections	*l* = −26→27

### Refinement

**Table:**

Refinement on *F*^2^	Secondary atom site location: difference Fourier map
Least-squares matrix: full	Hydrogen site location: inferred from neighbouring sites
*R*[*F*^2^ > 2σ(*F*^2^)] = 0.037	H atoms treated by a mixture of independent and constrained refinement
*wR*(*F*^2^) = 0.097	*w* = 1/[σ^2^(*F*_o_^2^) + (0.0489*P*)^2^ + 0.2287*P*] where *P* = (*F*_o_^2^ + 2*F*_c_^2^)/3
*S* = 1.07	(Δ/σ)_max_ = 0.001
3780 reflections	Δρ_max_ = 0.32 e Å^−^^3^
168 parameters	Δρ_min_ = −0.24 e Å^−^^3^
0 restraints	Absolute structure: Flack (1983), with 1584 Friedel pairs
Primary atom site location: structure-invariant direct methods	Flack parameter: −0.08 (7)

### Special details

**Table:**

Geometry. All e.s.d.'s (except the e.s.d. in the dihedral angle between two l.s. planes) are estimated using the full covariance matrix. The cell e.s.d.'s are taken into account individually in the estimation of e.s.d.'s in distances, angles and torsion angles; correlations between e.s.d.'s in cell parameters are only used when they are defined by crystal symmetry. An approximate (isotropic) treatment of cell e.s.d.'s is used for estimating e.s.d.'s involving l.s. planes.
Refinement. Refinement of *F*^2^ against ALL reflections. The weighted *R*-factor *wR* and goodness of fit *S* are based on *F*^2^, conventional *R*-factors *R* are based on *F*, with *F* set to zero for negative *F*^2^. The threshold expression of *F*^2^ > σ(*F*^2^) is used only for calculating *R*-factors(gt) *etc*. and is not relevant to the choice of reflections for refinement. *R*-factors based on *F*^2^ are statistically about twice as large as those based on *F*, and *R*-factors based on ALL data will be even larger.

### Fractional atomic coordinates and isotropic or equivalent isotropic displacement parameters (Å^2^)

**Table:**

	*x*	*y*	*z*	*U*_iso_*/*U*_eq_	
S1	−0.25429 (6)	0.29169 (3)	0.213725 (19)	0.02077 (10)	
O1	0.31886 (18)	0.26735 (11)	0.06564 (6)	0.0214 (2)	
N1	0.6103 (2)	0.39581 (13)	0.03384 (7)	0.0191 (3)	
N2	0.2245 (2)	0.50945 (11)	0.15475 (6)	0.0159 (2)	
N3	0.0861 (2)	0.40757 (13)	0.16284 (7)	0.0171 (2)	
N4	−0.1126 (2)	0.54025 (13)	0.23530 (7)	0.0181 (2)	
C1	0.6888 (2)	0.52394 (15)	0.05069 (8)	0.0170 (3)	
C2	0.8718 (2)	0.58576 (17)	0.02731 (8)	0.0206 (3)	
H2A	0.9661	0.5427	−0.0042	0.025*	
C3	0.9124 (2)	0.71447 (17)	0.05206 (8)	0.0217 (3)	
H3A	1.0386	0.7586	0.0374	0.026*	
C4	0.7740 (2)	0.78049 (15)	0.09758 (7)	0.0202 (3)	
C5	0.5903 (2)	0.71534 (15)	0.12059 (7)	0.0175 (3)	
H5A	0.4945	0.7585	0.1516	0.021*	
C6	0.5498 (2)	0.58673 (15)	0.09747 (7)	0.0157 (3)	
C7	0.3812 (2)	0.49169 (14)	0.11202 (7)	0.0158 (3)	
C8	0.4287 (2)	0.37009 (15)	0.06883 (8)	0.0175 (3)	
C9	−0.0900 (2)	0.42245 (14)	0.20479 (7)	0.0163 (3)	
C10	−0.2903 (2)	0.57322 (17)	0.28065 (9)	0.0246 (3)	
H10A	−0.3009	0.6702	0.2853	0.037*	
H10B	−0.2675	0.5333	0.3268	0.037*	
H10C	−0.4223	0.5383	0.2604	0.037*	
C11	0.8227 (3)	0.92103 (18)	0.12193 (9)	0.0288 (4)	
H11A	0.9767	0.9361	0.1204	0.043*	
H11B	0.7716	0.9327	0.1699	0.043*	
H11C	0.7511	0.9851	0.0912	0.043*	
H1N1	0.675 (4)	0.345 (2)	0.0091 (12)	0.030 (6)*	
H1N3	0.105 (3)	0.337 (2)	0.1401 (10)	0.022 (5)*	
H1N4	−0.027 (4)	0.598 (2)	0.2289 (11)	0.028 (6)*	

### Atomic displacement parameters (Å^2^)

**Table:**

	*U*^11^	*U*^22^	*U*^33^	*U*^12^	*U*^13^	*U*^23^
S1	0.01781 (16)	0.01833 (15)	0.02616 (18)	−0.00367 (14)	0.00191 (16)	0.00281 (13)
O1	0.0245 (5)	0.0189 (5)	0.0207 (5)	−0.0035 (4)	−0.0012 (4)	−0.0036 (4)
N1	0.0210 (6)	0.0189 (6)	0.0173 (6)	0.0018 (5)	0.0025 (5)	−0.0022 (5)
N2	0.0162 (6)	0.0145 (5)	0.0171 (5)	−0.0021 (4)	−0.0010 (5)	0.0021 (4)
N3	0.0167 (6)	0.0147 (5)	0.0200 (6)	−0.0019 (5)	0.0022 (5)	−0.0016 (4)
N4	0.0145 (5)	0.0195 (6)	0.0204 (6)	−0.0004 (5)	0.0015 (5)	−0.0009 (5)
C1	0.0175 (6)	0.0185 (6)	0.0149 (6)	0.0012 (5)	−0.0009 (5)	0.0020 (5)
C2	0.0171 (7)	0.0255 (7)	0.0191 (7)	0.0031 (6)	0.0023 (5)	0.0038 (6)
C3	0.0162 (6)	0.0277 (7)	0.0213 (7)	−0.0036 (6)	−0.0010 (5)	0.0069 (6)
C4	0.0211 (7)	0.0220 (6)	0.0174 (6)	−0.0062 (6)	−0.0035 (5)	0.0031 (5)
C5	0.0180 (6)	0.0185 (6)	0.0161 (6)	−0.0013 (6)	−0.0002 (5)	0.0003 (5)
C6	0.0148 (6)	0.0184 (6)	0.0140 (6)	0.0005 (5)	−0.0001 (5)	0.0013 (5)
C7	0.0167 (6)	0.0164 (6)	0.0143 (6)	−0.0005 (5)	−0.0021 (5)	−0.0008 (5)
C8	0.0197 (7)	0.0175 (6)	0.0154 (6)	0.0015 (5)	−0.0018 (5)	−0.0015 (5)
C9	0.0151 (6)	0.0170 (6)	0.0169 (6)	0.0004 (5)	−0.0026 (5)	0.0019 (5)
C10	0.0195 (7)	0.0294 (7)	0.0251 (7)	0.0036 (6)	0.0031 (6)	−0.0041 (6)
C11	0.0322 (8)	0.0273 (8)	0.0271 (8)	−0.0129 (7)	−0.0012 (7)	−0.0017 (7)

### Geometric parameters (Å, º)

**Table:**

S1—C9	1.6781 (15)	C2—H2A	0.9500
O1—C8	1.2419 (18)	C3—C4	1.398 (2)
N1—C8	1.348 (2)	C3—H3A	0.9500
N1—C1	1.414 (2)	C4—C5	1.398 (2)
N1—H1N1	0.81 (2)	C4—C11	1.516 (2)
N2—C7	1.2920 (19)	C5—C6	1.388 (2)
N2—N3	1.3510 (17)	C5—H5A	0.9500
N3—C9	1.3749 (19)	C6—C7	1.452 (2)
N3—H1N3	0.84 (2)	C7—C8	1.503 (2)
N4—C9	1.3259 (19)	C10—H10A	0.9800
N4—C10	1.4520 (19)	C10—H10B	0.9800
N4—H1N4	0.80 (2)	C10—H10C	0.9800
C1—C2	1.381 (2)	C11—H11A	0.9800
C1—C6	1.400 (2)	C11—H11B	0.9800
C2—C3	1.399 (2)	C11—H11C	0.9800
			
C8—N1—C1	110.85 (13)	C5—C6—C1	120.52 (14)
C8—N1—H1N1	127.0 (16)	C5—C6—C7	133.10 (14)
C1—N1—H1N1	122.0 (16)	C1—C6—C7	106.37 (13)
C7—N2—N3	117.30 (12)	N2—C7—C6	125.90 (13)
N2—N3—C9	120.18 (12)	N2—C7—C8	127.65 (13)
N2—N3—H1N3	119.1 (15)	C6—C7—C8	106.44 (12)
C9—N3—H1N3	120.6 (15)	O1—C8—N1	127.21 (14)
C9—N4—C10	123.25 (13)	O1—C8—C7	126.20 (13)
C9—N4—H1N4	120.3 (16)	N1—C8—C7	106.59 (13)
C10—N4—H1N4	116.4 (16)	N4—C9—N3	116.09 (13)
C2—C1—C6	121.61 (15)	N4—C9—S1	125.91 (11)
C2—C1—N1	128.67 (15)	N3—C9—S1	118.00 (11)
C6—C1—N1	109.72 (13)	N4—C10—H10A	109.5
C1—C2—C3	117.20 (15)	N4—C10—H10B	109.5
C1—C2—H2A	121.4	H10A—C10—H10B	109.5
C3—C2—H2A	121.4	N4—C10—H10C	109.5
C4—C3—C2	122.33 (14)	H10A—C10—H10C	109.5
C4—C3—H3A	118.8	H10B—C10—H10C	109.5
C2—C3—H3A	118.8	C4—C11—H11A	109.5
C3—C4—C5	119.23 (14)	C4—C11—H11B	109.5
C3—C4—C11	120.45 (14)	H11A—C11—H11B	109.5
C5—C4—C11	120.31 (15)	C4—C11—H11C	109.5
C6—C5—C4	119.07 (14)	H11A—C11—H11C	109.5
C6—C5—H5A	120.5	H11B—C11—H11C	109.5
C4—C5—H5A	120.5		
			
C7—N2—N3—C9	−176.69 (13)	N3—N2—C7—C6	−178.16 (13)
C8—N1—C1—C2	178.42 (15)	N3—N2—C7—C8	0.6 (2)
C8—N1—C1—C6	−1.59 (17)	C5—C6—C7—N2	−2.0 (3)
C6—C1—C2—C3	−0.3 (2)	C1—C6—C7—N2	177.54 (14)
N1—C1—C2—C3	179.68 (14)	C5—C6—C7—C8	178.97 (15)
C1—C2—C3—C4	−1.1 (2)	C1—C6—C7—C8	−1.48 (15)
C2—C3—C4—C5	1.3 (2)	C1—N1—C8—O1	179.56 (15)
C2—C3—C4—C11	−178.82 (15)	C1—N1—C8—C7	0.58 (16)
C3—C4—C5—C6	−0.1 (2)	N2—C7—C8—O1	2.6 (2)
C11—C4—C5—C6	−179.99 (14)	C6—C7—C8—O1	−178.43 (14)
C4—C5—C6—C1	−1.2 (2)	N2—C7—C8—N1	−178.43 (14)
C4—C5—C6—C7	178.28 (15)	C6—C7—C8—N1	0.57 (16)
C2—C1—C6—C5	1.5 (2)	C10—N4—C9—N3	179.39 (14)
N1—C1—C6—C5	−178.51 (13)	C10—N4—C9—S1	−1.4 (2)
C2—C1—C6—C7	−178.14 (13)	N2—N3—C9—N4	0.19 (19)
N1—C1—C6—C7	1.87 (16)	N2—N3—C9—S1	−179.14 (10)

### Hydrogen-bond geometry (Å, º)

*Cg*2 is the centroid of the C1–C6 ring.

**Table:**

*D*—H···*A*	*D*—H	H···*A*	*D*···*A*	*D*—H···*A*
N1—H1*N*1···O1^i^	0.81 (2)	2.03 (2)	2.8319 (17)	171 (2)
N3—H1*N*3···O1	0.84 (2)	2.079 (19)	2.7525 (17)	136.9 (17)
N4—H1*N*4···S1^ii^	0.80 (2)	2.85 (2)	3.5538 (13)	148.5 (19)
C3—H3*A*···*Cg*2^iii^	0.95	2.62	3.4165 (16)	142

Symmetry codes: (i) *x*+1/2, −*y*+1/2, −*z*; (ii) −*x*, *y*+1/2, −*z*+1/2; (iii) *x*+1/2, −*y*+3/2, −*z*.
